# Beyond the Usual Suspects: Ethylene Glycol Poisoning Complicated by Rare Neurological Sequelae

**DOI:** 10.7759/cureus.57868

**Published:** 2024-04-08

**Authors:** Preetham Ezhilarasu, Ragunanthanan Srinivasan

**Affiliations:** 1 Internal Medicine, Madras Medical College and Rajiv Gandhi Government General Hospital, Chennai, IND

**Keywords:** cerebellar ataxia, haemodialysis (hd), sudden sensorineural hearing loss (ssnhl), facial nerve paralysis, acute kidney failure, ethylene glycol poisoning, neurologic complications

## Abstract

Ethylene glycol poisoning is a known clinical entity with established diagnostic and management protocols. However, instances presenting with rare neurological complications pose diagnostic challenges and necessitate prompt recognition and intervention. This report details the case of ethylene glycol poisoning in a 38-year-old male patient who initially presented with a history of brake oil consumption at his residence, followed by a delayed presentation with vomiting, abdominal pain, and reduced urine output, and subsequently developed unusual neurological sequelae, including unsteadiness, hearing difficulties, and an inability to close his eyes. Diagnostic assessment revealed cerebellar ataxia with bilateral sensory-neural hearing loss and facial nerve palsy. The patient was subsequently managed primarily for ethylene glycol poisoning, with conservative management for the neurological sequelae, and improved with no residual deficits. This case underscores the importance of promptly managing ethylene poisoning to prevent complications and sequelae as well as reduce morbidity for patients.

## Introduction

Despite its innocuous appearance and taste, ethylene glycol is a highly toxic compound. Found in everyday items like antifreeze, air conditioning systems, and windscreen de-icing fluid [1], its rapid absorption into the bloodstream leads to toxicity primarily from its by-products rather than the compound itself [2]. Poisoning with ethylene glycol can occur intentionally for intoxication, suicidal ideation, or accidentally.

Clinical manifestations typically progress through three phases. Phase 1 (0.5-12 hours): CNS symptoms including inebriation, ataxia, seizures, coma, and potentially death. Gastrointestinal irritation may cause nausea and vomiting. Phase 2 (12-24 hours): accumulation of organic acids leading to cardiopulmonary syndrome with symptoms like tachycardia, hypertension, tachypnea, and pulmonary edema. Phase 3 (24-72 hours): renal failure due to osmotic damage and calcium oxalate accumulation in the kidneys, with metabolic acidosis persisting throughout [3].

Additionally, severe cases may exhibit a fourth phase characterized by neurological sequelae, including delayed cranial neuropathies, cerebral edema, seizures, increased intracranial pressure, stroke-like symptoms, diaphragmatic paralysis, sensory radiculopathies, and autonomic nervous system dysfunction [3].

These neurological complications highlight the multifaceted nature of ethylene glycol toxicity, necessitating thorough evaluation and management. We report a unique combination of cerebellar ataxia, bilateral sensory-neural hearing loss, and bilateral lower motor neuron facial nerve palsy as delayed sequelae of ethylene glycol.

## Case presentation

A 38-year-old male of Indian ethnicity reportedly consumed 250 ml of brake oil, containing ethylene glycol, in a suicidal attempt at his residence. Three days later, the patient developed complaints of abdominal pain, vomiting, and reduced urine output. It was not until the fifth day that the patient disclosed his ingestion to family members, prompting his admission to the emergency room.

During the initial evaluation, the patient appeared conscious and cooperative, albeit with signs of physiological distress: tachycardia (106/min), tachypnea (26/min), normal oxygen saturation (99% in room air), blood pressure of 144/84 mmHg, and capillary blood glucose level of 106 mg/dl. A comprehensive systemic examination revealed no overt abnormalities.

Laboratory investigations unveiled significant findings, including a high anion gap, metabolic acidosis, acute kidney injury, mild hyponatremia, transaminitis, and a normal serum osmolal gap (Table 1).

**Table 1:** Laboratory workup on admission

Blood work	Value	Reference range
Hemoglobin (Hb)	13.2 mg/dl	13.5-15.5 mg/dl
Total leukocyte count	12,500 cells/mm3	4,000-11,000 cells/mm3
Platelets	187,000 cells/mm3	150,000-450,000 cells/mm3
Serum urea	64.2 mg/dl	10-40 mg/dl
Serum creatinine	5.1 mg/dl	0.3-1.0 mg/dl
Serum sodium (Na)	128 Meq/L	135-145 Meq/L
Serum potassium (K)	3.8 Meq/L	3.5-5 Meq/L
Serum corrected calcium (Ca)	8.9 mg/dl	8.4-10.4 mg/dl
Serum uric acid	9.1 mg/dl	4.5-6.5 mg/dl
Total bilirubin	0.6 mg/dl	0.1-1.2 mg/dl
Aspartate transaminase (AST)	146 units/L	10-40 units/L
Alanine transaminase (ALT)	114 units/L	10-40 units/L
pH	7.32	7.35-7.45
Serum bicarbonate	17.6 Meq/L	24-28 Meq/L
Serum chloride	91 Meq/L	96-106 Meq/L
Anion gap	19.4 Meq/L	10-14 Meq/L
Measured serum osmolality	277 mOsm/kg	275-295 mOsm/kg
Calculated serum osmolality	272 mOsm/kg	275-295 mOsm/kg
Osmolal gap	5 mOsm/kg	<10 mOsm/kg

Urine screens for common toxins, including paracetamol, salicylates, and paraquat, were negative, while serum ethylene glycol levels were measured at 7 mg/dl. Additional diagnostic tests, such as the electrocardiogram (ECG), chest radiograph, and abdominal ultrasonogram, returned unremarkable results.

The patient received immediate treatment with an intravenous bolus dose of fomepizole (1 g), thiamine (200 mg), and pyridoxine (50 mg). The subsequent monitoring revealed no improvement in urine output over the following three hours, prompting the initiation of urgent hemodialysis. Continuous treatment involved maintenance doses of fomepizole (650 mg every six hours), oral vitamin supplements, and daily hemodialysis for the first two cycles, transitioning to alternate-day hemodialysis thereafter. The patient remained anuric until day five of hospitalization, with a peak creatinine level of 12.3 mg/dl, before gradually showing signs of improvement in both renal function and urine output.

On day 10 of hospitalization, the patient complained of difficulty closing both his eyes. On examination, the patient had weakness in eye closure, loss of nasolabial folds, and an inability to smile. The remainder of the neurological examination was within normal limits. A diagnosis of bilateral lower motor neuron-type facial nerve palsy was made, and appropriate investigations were scheduled. On day 13 of hospitalization, the patient complained of dizziness, difficulty hearing, and difficulty maintaining balance. Subsequent physical examination showed signs suggestive of cerebellar impairment, including dysdiadochokinesia, impaired finger-nose test, impaired heel-shin, and tandem walking, but no dysarthria (Videos 1, 2). The examination also showed signs of vestibulocochlear nerve impairment.

**Video 1:** Demonstrating impaired finger nose test

**Video 2:** Demonstrating impaired heel-shin test

**Figure 1:**
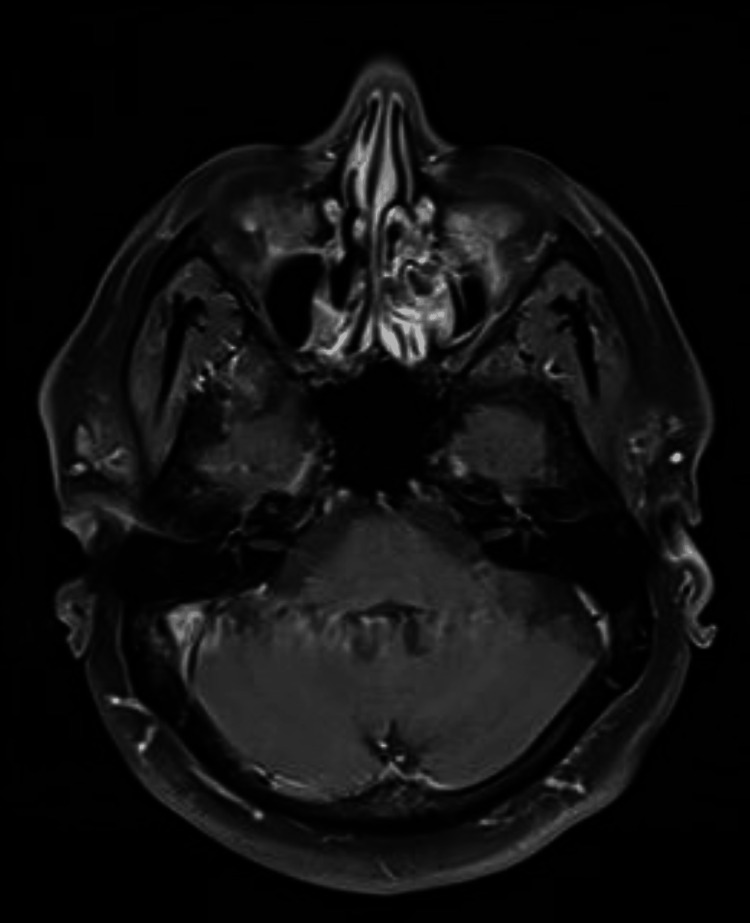
MRI T1-weighted bulbar axial cut

**Figure 2:**
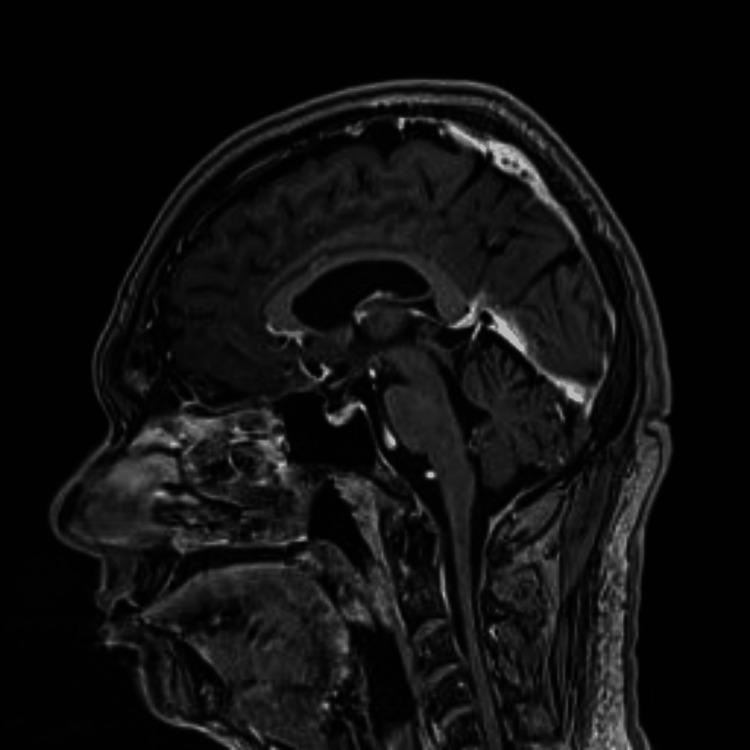
MRI T1-weighted midline sagittal cut

MRI with gadolinium contrast was normal (Figures 1, 2). A nerve conduction study (NCS) of the face showed findings consistent with demyelinating neuropathy. Pure tone audiometry revealed mixed hearing loss with sensi-neural components at higher frequencies. The patient was managed conservatively for his neurological symptoms with physiotherapy and programmed exercise.

Following the completion of 14 cycles of hemodialysis, the patient was discharged on day 38 of hospitalization, having achieved a nadir creatinine level of 5.6 mg/dl. Over the subsequent three months, gradual neurological improvement led to a complete recovery. Creatinine levels stabilized at a baseline of 3.2 mg/dl, managed conservatively.

## Discussion

Our patient presented with a unique combination of neurological sequelae; its temporal association with ethylene glycol (EG) consumption as well as consistent neurological investigation provide compelling evidence of EG as the etiology. While ataxia has been documented in previous studies [4,5], the occurrence of cerebellar ataxia specifically has not been reported. Additionally, our patient exhibited cranial nerve palsies, including bilateral facial and vestibulocochlear nerve involvement, consistent with common neurological sequelae [6,7].

As a consequence of delayed presentation, on the fifth day post-EG consumption, our patient was already in phase 3 of intoxication. This accounts for the normal serum osmolal gap [8] and relatively low serum EG levels. Although fomepizole was administered as a precautionary measure, its efficacy in this scenario is debatable due to the delayed presentation [9].

The onset of neurological sequelae aligns with timelines observed in previous studies [6,7]. While the exact mechanism remains incompletely understood, it is believed to involve the deposition of oxalate crystals within CNS blood vessels, leading to endothelial injury [10,11]. Notably, the cerebellar ataxia observed in our case presents a distinct feature. With the absence of dysarthria, normal neuroimaging findings, and intact sensory and motor extremities, the likely site of involvement appears to be the cerebellum's connection to the brainstem and higher centers. This conclusion is supported by research demonstrating EG-induced brainstem involvement and white matter tract damage on MRI [12].

Another noteworthy aspect is the presence of demyelinating neuropathy in nerve conduction studies, contrary to the anticipated axonal pattern [13]. Possible explanations for this discrepancy include a recovering lesion [14] or procedural errors during the study.

While our patient experienced complete neurological recovery over three months with conservative management, renal dysfunction persisted, as evidenced by elevated creatinine levels. This recovery pattern mirrors observations in existing literature [7,15].

## Conclusions

In conclusion, our case underscores the diverse neurological manifestations of EG poisoning, including the rare occurrence of cerebellar ataxia. This case further emphasizes the need to diagnose EG poisoning promptly to prevent complications. The patient's neurological symptoms showed significant improvement over time with conservative management. However, renal dysfunction persisted. This case highlights the importance of considering EG toxicity in patients, even in the absence of classic metabolic disturbances. Further research is warranted to elucidate the underlying mechanisms and optimize treatment strategies for EG poisoning-associated neurological complications.
